# Seroepidemological investigation of *Toxoplasma gondii* and *Trichinella* spp. in pigs reared by tribal communities and small-holder livestock farmers in Northeastern India

**DOI:** 10.1371/journal.pone.0298357

**Published:** 2024-02-20

**Authors:** A. A. P. Milton, Samir Das, S. Ghatak, K. Srinivas, G. Bhuvana Priya, M. Angappan, M. C. B. Prasad, L. Wahlang, Blessa Sailo, Lalhruaipuii Lalhruaipuii, Mahak Singh, G. B. Garam, A. Sen

**Affiliations:** 1 ICAR Research Complex for NEH Region, Umiam, Meghalaya, India; 2 College of Agriculture, Central Agricultural University (Imphal), Kyrdemkulai, Meghalaya, India; 3 Department of Animal Husbandry, Veterinary & Dairy Development, Nirjuli, Arunachal Pradesh, India; Kerman University of Medical Sciences, ISLAMIC REPUBLIC OF IRAN

## Abstract

*Toxoplasma gondii* and *Trichinella* spp. are critical tissue-dwelling foodborne zoonotic parasites associated with pork consumption and pig rearing. Despite being a major pig-rearing region in the country, Northeastern India has not undergone any investigation regarding the presence of *T*. *gondii* and *Trichinella* spp. in pigs. Therefore, this study aims to determine the seroprevalence of *T*. *gondii* and *Trichinella* spp. and identify associated risk factors in pigs reared by tribal communities and small-holder livestock farmers in the northeastern region of India. In a cross-sectional serological survey, 400 pigs from 400 households across five northeastern states of India underwent testing for the seroprevalence of porcine toxoplasmosis and trichinellosis. Serum samples (80 from each state) were analyzed using commercially available ELISA assays. Data on backyard farm characteristics and various management aspects were collected, and risk factors linked with prevalence were analyzed through univariate and multivariate logistic regression analysis. The findings revealed that the apparent and true prevalence of anti-*T*. *gondii* antibodies were 45% (40.12–49.88, 95% CI) and 45.7% (40.7–50.69, 95% CI), respectively. As for anti- *Trichinella* antibodies, both the apparent and true prevalence were 0.75% (-0.1–1.6, 95% CI). The univariate and multivariate analyses indicated that age above 24 months (OR 7.20, 95% CI 2.45–23.71), exposure to cats (OR = 5.87, 95% CI 2.55–14.05), and farms operating for breeding purposes (OR = 5.60, 95% CI 3.01–11.04) were significant risk factors associated with the seroprevalence of *T*. *gondii*. This study marks the initial documentation of the seroprevalence of *T*. *gondii* and *Trichinella* spp. in pigs reared by tribal communities in Northeastern India. The results emphasize the significance of these parasites as foodborne zoonotic threats in the region, potentially posing substantial public health risks, especially within tribal and rural communities. The insights derived from this research could be valuable in formulating targeted preventive and control strategies against *T*. *gondii* and *Trichinella* spp. in pigs, not only in this region but also in areas with similar rearing practices.

## Introduction

Zoonotic diseases are considered a significant threat to public health, social stability and the world economy [1, 2]. It is estimated by the World Health Organization (WHO) that every year, approximately one billion illnesses and millions of human fatalities emerge globally due to zoonotic diseases [3]. Over 60% of the newly emerging infectious diseases reported globally stem from zoonotic sources, and about 75% of the most recent 30 emerging human pathogens have their roots in animal populations [1]. Zoonotic infections resulting from parasite transmission are widespread and common. Parasitic zoonotic infections are transmitted through the consumption of contaminated food and water, close contact with animals, and exposure to infected soil. Despite imposing a significant global health burden on individuals and exerting substantial financial pressure on the livestock sector, most parasitic zoonoses are considered neglected diseases [4]. In India, many such parasitic zoonoses are endemic and reemerge in regular outbreaks [5, 6]. Since ancient times, swine have been implicated as hosts of zoonotic parasites, including *T*. *gondii* and *Trichinella* spp. [7].

*T*. *gondii* is an obligate coccidian intracellular parasite with the capability to infect a broad spectrum of warm-blooded vertebrates, including humans, and continues to be a major public health concern. Antibodies against this parasite are carried by more than one-third of the world’s human population [8, 9]. It has been reported that approximately 24% of all deaths attributed to foodborne pathogens are caused by *T*. *gondii* alone [10]. Cats serve as key definitive hosts shedding resistant oocysts in their faeces [9]. Pigs play a crucial role as an intermediate host, acquiring infections through contaminated feed, water, and rodent carcasses that may contain viable tissue cysts of *T*. *gondii*.

*Trichinella* spp. is a foodborne zoonotic helminth infecting human and a wide range of animals, including domestic pigs, wild boars, rats, horses, etc [11]. This disease is recognized to affect approximately ten thousand individuals annually, with a mortality rate of around 0.2% [12]. Trichinellosis holds significance not only for its zoonotic potential but also for its substantial influence on swine production, food safety and international trade [13]. Omnivores and carnivores, including those with predating, scavenging, and cannibalistic tendencies, can serve as primary reservoirs for *Trichinella* spp. Pigs, in particular, have a significant role as a reservoir and are a common source of human trichinellosis. As a result, human *Trichinella* infections are typically concentrated in regions where pork consumption is widespread [11].

In the present study, the seroprevalence of *T*. *gondii* and *Trichinella* spp. in pigs reared by tribal communities and smallholder farmers in five states (Arunachal Pradesh, Meghalaya, Manipur, Nagaland, and Mizoram) in northeastern India was aimed to be determined. This region houses 46.8% of the pig population of India and shares porous international borders with four countries. Furthermore, the risk factors associated with seroprevalence were analysed. The data and knowledge generated from this investigation are considered crucial for developing and implementing evidence-based surveillance systems and effective one health approach-based control programs for *T*. *gondii* and *Trichinella* spp. in the pig and human populations of the region. Additionally, this survey is recognized as the first, to our knowledge, to record the seroprevalence of *T*. *gondii* in backyard pigs in Northeastern India.

## Materials and methods

### Ethical approval

The ethical approval for this study was granted by the Institutional Animal Ethics Committee registered with the Committee for Control and Supervision of Experiments on Animals (CCSEA) and Institutional Research Committee (IXX14426) of the ICAR Research Complex for North Eastern Hill Region, Umiam, Meghalaya. The pigs during blood collection were restrained by the qualified veterinarian. The questionnaire related to risk factors were completed and a verbal informed consent was obtained from the animal owners and the same was documented.

### Study design and site

Between March 2019 and October 2020, a cross-sectional study was undertaken. The primary pig-rearing zone of India, which accounts for 46.8% of the total porcine population, was selected for this investigation. The study was conducted in five north-eastern states of India: (Arunachal Pradesh 28.2180° N, 94.7278° E, Meghalaya (25.4670° N, 91.3662° E, Manipur 24.6637° N, 93.9063° E, Mizoram (23.1645° N, 92.9376° E and Nagaland (26.1584° N, 94.5624° E) of India. These states are characterized by hilly terrain and are primarily inhabited by tribal communities, with varying levels of diversity observed within their communities. In Arunachal Pradesh, samples were collected from villages situated in six districts, namely Kamle, Upper Subansiri, East Siang, Lower Dibang Valley, Namsai, and Changlang. In Meghalaya, samples were collected from three districts: West Khasi Hills, East Khasi Hills, and West Jaintia Hills. In Manipur, the covered districts were East Imphal and West Imphal. In Mizoram and Nagaland, the covered districts were Kolasib and Serchhip, and Dimapur, respectively.

### Sampling plan and population

In the 20^th^ Census conducted in 2019, India’s total pig population was recorded as 9.06 million. The focus of our study was on five specific states, collectively accounting to 21% of the country’s pig population, amounting to 1,910,242 pigs. A total of 400 serum samples were utilized in this study, collected from five states with an equal distribution of 80 samples from each state as reported previously [14]. The required sample size for toxoplasmosis, computed using Statulator [15], was determined to be 384 (95% confidence level; 5% margin of error). A toxoplasmosis seroprevalence of 48% was assumed based on the findings of a prior study conducted in North India [16]. Similarly, for trichinellosis, considering the previously reported prevalence of 0.69% [17], the required sample size was 87. Given that the majority of pigs are raised in small-holder systems, an opportune sampling approach was employed by collecting one serum sample from a pig in each household or farm. After suitable precautions were taken, blood samples were obtained. In adults and piglets, samples were collected from the ear vein and external jugular vein/anterior vena cava, respectively. The collection sites were cleaned with alcohol before blood collection. Subsequently, the blood was allowed to coagulate, and serum was separated through centrifugation at 3000 rpm for 10 minutes [14].

### Questionnaire

Following the collection of blood samples, brief interviews were conducted with animal owners to complete a questionnaire. The purpose of this questionnaire was to acquire information about farm characteristics and various management practices, with the goal of identifying potential risk factors associated with *T*. *gondii* and *Trichinella* spp. infections. The survey encompassed a variety of questions, including age, gender, housing and rearing system, abortion history in pigs and handlers, feed source, origin of stock, production system, exposure to cats, rodents and birds as well as management practices such as disinfection, deworming, etc.

### Serological testing

The screening for the presence of *T*. *gondii* and *Trichinella* spp. antibodies involved the use of commercially available ELISA kits, namely the PrioCHECK^®^ Porcine *Toxoplasma* Ab Kit and the PrioCHECK Porcine *Trichinella* Ab Strip Kit, both manufactured by Prionics, Switzerland. The tests were executed in accordance with the manufacturer’s instructions. To test for *T*. *gondii* antibodies, serum samples were diluted at a ratio of 1:100 and placed to microtitre plates pre-coated with cell culture-derived *T*. *gondii* tachyzoite antigen. The *Toxoplasma* Ab Kit, as per the manufacturer’s information, exhibits a blood-plasma sensitivity of 98% and a specificity of 100%. The optical density (OD) was measured at 450 nm (with a reference filter at 620 nm), and the test results were interpreted by calculating a percentage of positivity (PP) value for each sample. This PP value was determined relative to the OD of the positive control using the formula: PP Sample = OD450 nm Sample / OD450 nm Positive Control x 100. In accordance with manufacturer’s guidelines, a PP value ≥ 20 was considered positive, while PP values below 20 were considered negative. For the detection of *Trichinella* spp. antibodies, serum samples were diluted at a ratio of 1:50 and applied to microtiter plates coated with the E/S antigen of *T*. *spiralis*. According to manufacturer’s data, the PrioCHECK^®^ *Trichinella* Ab ELISA demonstrated a sensitivity and specificity of 100%. Results meeting or exceeding the manufacturer’s cut-off of 15% PP were classified as ELISA positive.

### Statistical analysis

Confidence intervals (CI) for the seroprevalence values were established at a 95% confidence level. The Rogan-Gladen estimator, as described by Rogan and Gladen [18] was employed to estimate the true prevalence. In the study, univariate analysis was conducted to explore potential associations between various variables and seroprevalence. The Likelihood Ratio Chi-Squared test (LRT) was used to assess these associations. Additionally, for variables such as breed, abortion/still birth/ocular disease history in the family, and cat exposure, which did not meet the assumptions of the Chi-Squared test, Fisher’s t-test was employed. A statistically significant result was defined as a *p*-value less than 0.05. The strength of these associations was measured computing odds ratios with 95% CI (confidence intervals). For multivariate regression analysis, all variables were included and a forward selection step-wise approach based on the LRT was used to determine the best model. Throughout this process, it was ensured that Variance Inflation Factors (VIF) remained below 5 to avoid issues of multicollinearity. The goodness-of-fit of the final model was assessed using the LRT statistic, and residuals were examined. All statistical analyses were conducted using R Studio software version 4.2.2, with the assistance of packages such as "epiDisplay," "vcd," and "car."

## Results

### Descriptive analysis and features of the study population

An equal number of samples (n = 80) were collected from all five states in northeastern India, totalling 400 samples, covering 400 households. The mean age of the pig population was ≈11.1 months, with ages ranging from 0 to over 24 months. To provide a broad overview of the age distribution within the study population, age was categorized into four groups: 0–6 months, 7–12 months, 13–24 months, and over 24 months. A significant majority fell into the "young" category, with pigs aged 0–6 months accounting for 23.5% (94/400) of the total sample. Pigs in the 7–12 months age group represented 43.75% (175/400) of the population. Those in the 13–24 months age group constituted 22.5% (90/400) of the sample, while pigs over 24 months old made up 10.25% (41/400) of the total. The population consisted of 57.5% female pigs and 42.5% male pigs. Crossbred pigs were the most common, comprising 78.75% (315 out of 400), while exotic breeds accounted for only 2%, and local pigs constituted 19.25%. The majority of households raised fewer than 10 pigs (77.75%), while 15.5% raised between 11 and 20 pigs, and only 6.75% raised more than 20 pigs. Farm sizes were categorized as small (1 to 10), medium (11 to 20), and large (more than 20) for analysis. Fattening purposes, following the all-in-all-out system, accounted for 49% of pigs, while 32.5% were raised for fattening purposes following the continuous cycle system, and 18.5% were raised for breeding purposes to produce piglets. Out of the 400 households participating in the serological survey, only 0.5% (2 out of 400) reported a history of abortion, stillbirth, or ocular disease in the family. The majority of pigs (63.25%) were reared using the confined housing system, as opposed to pigs reared in semi-confined conditions (17.5%) and non-confined conditions (19.25%). Concrete floors were present in 55.5% (222/400) of farms, while non-concrete floors were found in 44.5% (178/400) of farms. Regular deworming of pigs and disinfection of enclosures were reported by 40% (160/400) and 62.25% (249/400) respondents, respectively. Exposure to cats was recorded in 43.75% (175 out of 400) households, exposure to rodents was recorded in 43.75% (175 out of 400) households, and exposure to birds was recorded in 64.25% (257 out of 400) households. Concerning feed sources, 64.75% (259 out of 400) of households fed their pigs with commercial and household feed, 5.75% (23 out of 400) fed their pigs exclusively with household kitchen waste, while 29.5% (118 out of 400) fed their pigs only with swill feed obtained from restaurants and bakeries. Out of the 400 households that participated in the serological survey, 22.75% (91 out of 400) did not have lavatories, while 77.25% (309 out of 400) of the sampled animals belonged to households with lavatories. Table 1 presents a summary of the different demographic characteristics and seroprevalence data.

**Table 1:** Univariate analysis of associated risk factors for seropositivity of anti-*T*. *gondii* antibodies.

Variable	Descriptive	Positive	OR (95% CI)	p-value
	Yes/Total (%)	Yes/Total (%, 95% CI)		
**State**				< 0.0001
Meghalaya	80/400 (20)	35/80 (43.75, 32.68–55.30)	2.68 (1.36–5.40)	0.0049
Arunachal Pradesh	80/400 (20)	18/80 (22.50, 13.91–33.21)	Reference	Reference
Manipur	80/400 (20)	13/80 (16.25, 8.95–26.18)	0.67 (0.30–1.47)	0.3190
Nagaland	80/400 (20)	66/80 (82.50, 72.38–90.09)	16.23 (7.66–36.65)	< 0.0001
Mizoram	80/400 (20)	48/80 (60.00, 48.44–70.80)	5.17 (2.63–10.50)	< 0.0001
**Age (in months)**				< 0.0001
0–6	94/400 (23.5)	29/94 (30.85, 21.73–41.22)	Reference	Reference
7–12	175/400 (43.75)	71/175 (40.57, 33.23–48.24)	1.53 (0.90–2.63)	0.1168
13–24	90/400 (22.5)	45/90 (50.00, 39.27–60.73)	2.24 (1.23–4.12)	0.0086
over 24	41/400 (10.25)	35/41 (85.37, 70.83–94.43)	13.07 (5.27–37.69)	< 0.0001
**Gender**				0.3599
Male:	170/400 (42.5)	72/170 (42.35, 34.82–50.15)	0.83 (0.56–1.24)	0.3600
Female:	230/400 (57.5)	108/230 (46.96, 40.37–53.63)	Reference	Reference
**Farm altitude**				0.0115
Lowland/ Valley	140/400 (35)	75/140 (53.57, 44.95–62.03)	Reference	Reference
Highland/ Hilly	260/400 (65)	105/260 (40.38, 34.37–46.62)	0.59 (0.39–0.89)	0.0118
**Stock replacement sources**				0.0008
Bred on own farm	92/400 (23)	47/92 (51.09, 40.44–61.66)	1.02 (0.62–1.68)	0.9265
Purchased from live market	110/400 (27.5)	33/110 (30.00, 21.63–39.48)	0.42 (0.25–0.68)	0.0005
Acquired from breeder/ other farm	198/400 (49.5)	100/198 (50.51, 43.33–57.67)	Reference	Reference
**Breed types**				0.0070
Exotic	8/400 (2)	1/8 (12.50, 00.32–52.65)	0.19 (0.01–1.10)	0.1259
Local	77/400 (19.25)	45/77 (58.44, 46.64–69.57)	1.90 (1.15–3.17)	0.0128
Crossbred	315/400 (78.75)	134/315 (42.54, 37.01–48.21)	Reference	Reference
**Farm size**				0.0008
Small (1 to 10)	311/400 (77.75)	154/311 (49.52, 43.83–55.22)	1.42 (0.65–3.26)	0.3830
Medium (11–20)	62/400 (15.50)	15/62 (24.19, 14.22–36.74)	0.46 (0.18–1.23)	0.1180
Large (> 20)	27/400 (6.75)	11/27 (40.74, 22.39–61.20)	-	-
**Production methods**				< 0.0001
All-in-all-out (for fattening)	196/400 (49)	85/196 (43.37, 36.32–50.62)	Reference	Reference
Continuous cycle (for fattening)	130/400 (32.5)	35/130 (26.92, 19.52–35.40)	0.48 (0.30–0.77)	0.0028
Breeding	74/400 (18.5)	60/74 (81.08, 70.30–89.25)	5.60 (3.01–11.04)	< 0.0001
**History of Abortion/ still birth in pigs**				0.0174
Yes	67/400 (16.75)	39/67 (58.21, 45.52–70.15)	1.90 (1.12–3.25)	0.0184
No	333/400 (83.25)	141/333 (42.34, 36.97–47.85)	Reference	Reference
**History of abortion/ still birth/ ocular disease in family**				1.000
Yes	2/400 (0.5)	1/2 (50.00, 1.26–98.74)	1.22 (0.49–31.08)	0.8869
No	398/400 (99.5)	179/398 (44.97, 40.01–50.01)	Reference	Reference
**Housing system**				< 0.0001
Confined	253/400 (63.25)	136/253 (53.75, 47.40–60.02)	Reference	Reference
Semi-confined	70/400 (17.5)	17/70 (24.29, 14.83–36.01)	0.54 (0.31–0.92)	0.0257
Non-confined	77/400 (19.25)	27/77 (35.06, 24.53–46.78)	0.24 (0.13–0.43)	< 0.0001
**Type of flooring**				0.2170
Concrete	222/400 (55.5)	106/222 (47.75, 41.02–54.54)	Reference	Reference
Non-concrete	178/400 (44.5)	74/178 (41.57, 34.25–49.18)	0.78 (0.52–1.16)	0.2180
**Disinfection**				0.5272
Yes	249/400 (62.25)	109/249 (43.78, 37.52–50.18)	0.88 (0.58–1.32)	0.5270
No	151/400 (37.75)	71/151 (47.02, 38.86–55.30)	Reference	Reference
**Exposure to cats**				0.0609
Yes	175/400 (43.75)	88/175 (50.29, 42.64–57.92)	1.46 (0.98–2.18)	0.0613
No	225/400 (56.25)	92/225 (40.89, 34.40–47.62)	Reference	Reference
**Exposure to rodents**				0.0609
Yes	175/400 (43.75)	88/175 (50.29, 42.64–57.92)	1.46 (0.98–2.18)	0.0613
No	225/400 (56.25)	92/225 (40.89, 34.40–47.62)	Reference	Reference
**Exposure to birds**				0.0042
Yes	257/400 (64.25)	102/257 (39.69, 33.66–45.96)	0.54 (0.36–0.83)	0.0044
No	143/400 (35.75)	78/143 (54.55, 46.01–62.88)	Reference	Reference
**Sources of feed**				0.0236
Commercial and household sources	259/400 (64.75)	124/259 (47.88, 41.65–54.15)	Reference	Reference
Exclusively household kitchen waste	23/400 (5.75)	14/23 (60.87, 38.54–80.29)	1.69 (0.72–4.20)	0.2364
Swill (from restaurants and bakeries)	118/400 (29.5)	42/118 (35.59, 27.00–44.93)	0.60 (0.38–0.94)	0.0265
**Deworming**				< 0.0001
Yes	160/400 (40)	52/160 (32.50, 25.32–40.35)	0.42 (0.28–0.64)	< 0.0001
No	240/400 (60)	128/240 (53.33, 46.81–59.78)	Reference	Reference
**Availability of lavatories**				0.1477
Yes	309/400 (77.25)	133/309 (43.04, 37.45–48.77)	0.71 (0.44–1.13)	0.1480
No	91/400 (22.75)	47/91 (51.65, 40.93–62.26)	Reference	Reference

### Seroprevalence of anti-*T*. *gondii* and anti-*Trichinella* antibodies

The seroprevalence of *T*. *gondii* and *Trichinella* spp. infection in backyard pigs reared in Northeastern India was determined using commercial ELISA assays. Out of 400 samples, 180 samples (45%) tested positive for *T*. *gondii*, while only 3 samples (0.75%; 3/400) were found positive for *Trichinella* spp. infection. The apparent and true prevalence of anti-*T*. *gondii* antibodies were 45% (40.12–49.88, 95% CI) and 45.7% (40.7–50.69, 95% CI), respectively (Table 1). Similarly, the apparent and true prevalence of anti-*Trichinella* antibodies were 0.75% (-0.1–1.6, 95% CI) and 0.75% (-0.1–1.6, 95% CI), respectively. Anti-*T*. *gondii* antibodies were detected in 82.5%, 60%, 43.75%, 22.5% and 16.25% of samples collected from Nagaland, Mizoram, Meghalaya, Arunachal Pradesh and Manipur, respectively. *Trichinella* spp. antibodies were detected only in Manipur (2/80, 2.5%, 0.30–8.74, 95% CI) and Arunachal Pradesh (1/80, 1.25%, 0.03–6.77, 95% CI).

### Potential risk factors

Twenty variables including animal and farm characteristics, management practices and disease conditions in farmers, were examined through univariate analysis. Few variables demonstrated a significantly strong association with the occurrence of *T*. *gondii* antibodies (p ≤ 0.05). For multivariate analysis, all variables were included, revealing that four variables were significantly associated with the seroconversion outcome (p ≤ 0.05). The odds ratios (OR) with CI and p values for determining potential risk factors for *T*. *gondii* seropositivity using univariate and multivariate logistic regression models are presented in Tables 1 and 2, respectively. In univariate analysis, significant variables at the animal level included age, where animals aged between 13 to 24 months (OR = 2.24, 95% CI 1.23–4.12) and over 24 months (OR = 13.07, 95% CI 5.27–37.69) showed a significant association with *T*. *gondii* seropositivity. Additionally, local or non-descript breeds were more likely to be seropositive (OR = 1.90, 95% CI 1.15–3.17). Farm characteristics such as production methods revealed that farms operating for breeding purposes (OR = 5.60, 95% CI 3.01–11.04), were strongly associated with higher odds of *T*. *gondii* seropositivity (p < 0.0001) compared to fattening farms following all-in-all-out and continuous cycles. Farms with a history of abortion and stillbirth in pigs (OR = 1.90, 95% CI 1.12–3.25) had significantly higher odds of *T*. *gondii* infection than those without such history. Farms that purchased piglets from live markets had significantly lower odds of *T*. *gondii* infection compared to farms using piglets from their own farm (OR = 1.02, 95% CI 0.62–1.68). The final multivariate logistic regression model (Table 2) revealed two variables, namely age and exposure to cats, with a significantly stronger association with *T*. *gondii* infection. Pigs aged between 13 to 24 months (OR = 2.58, 95% CI 1.22–5.57) and pigs older than 24 months (OR = 7.20, 95% CI 2.45–23.71) demonstrated a significantly higher likelihood of *T*. *gondii* antibodies. Similarly, farms with exposure to cats (OR = 5.87, 95% CI 2.55–14.05) were significantly more likely to have *T*. *gondii* seropositivity compared to farms with no cat exposure. Univariate and multivariate analyses for *Trichinella* spp. infection were not conducted due to the low seropositivity rate of only 0.75% (3/400) recorded in the present study.

**Table 2:** Multivariate logistic regression model for the analysis of risk factors associated with *T*. *gondii* infection in pigs.

Variables	Adjusted Odds Ratio (95% Confidence Interval)	p-value	Estimate	SE (estimate)
**Constant**			0.1176	0.2917
**Age (in months)**				
0–6	Reference	Reference	Reference	Reference
7–12	1.68 (0.94–3.05)	0.0847	0.5174	0.3001
13–24	2.58 (1.22–5.57)	0.0143*	0.9482	0.3870
over 24	7.20 (2.45–23.71)	0.0006*	1.9742	0.5737
**Housing system**				
Confined	Reference	Reference	Reference	Reference
Semi-confined	0.42 (0.22–0.79)	0.0075*	-0.8664	0.3242
Non-confined	0.14 (0.07–3.0)	<0.0001*	-1.9424	0.3828
**Exposure to cats**				
Yes	5.87 (2.55–14.05)	<0.0001*	1.7701	0.4336
No	Reference	Reference	Reference	Reference
**Exposure to birds**				
Yes	0.14 (0.07–0.28)	<0.0001*	-1.9501	0.3612
No	Reference	Reference	Reference	Reference

*Statistically significant variables

## Discussion

In India, the seroprevalence of *T*. *gondii* has been investigated in various animals, including livestock, poultry, and captive wild species such as cattle [19, 20], chicken [21], Mithun [22], sheep [23], goats [24], as well as Asiatic lions and Royal Bengal tigers [25]. Thakur et al. [16] reported a seroprevalence of 48.3% (73/151) in pigs reared in Punjab. In a recent study, a seroprevalence of 24.7% was reported among pigs raised in various agroclimatic zones of the North Indian state of Haryana [26]. This study represents the first large-scale investigation covering five northeastern states of India. Moreover, the reported seroprevalence of *T*. *gondii* in our study (45%) falls within the range of previous reports [21, 26]. In this study, a *T*. *gondii* seroprevalence of 45% in pigs was observed. Notably, comparable results have been documented in studies employing the PrioCHECK^®^ ELISA kit. For instance, Basso et al. [27] reported a seroprevalence of 46.1% in pig sera in Switzerland using the PrioCHECK^®^ ELISA. Similarly, Macaluso et al. [28] conducted a study involving 115 naturally exposed and 12 experimentally infected pig sera, revealing a seroprevalence of 51.9% with the same kit. Additionally, a study in Italy by Pipia et al. [29] documented a *T*. *gondii* seroprevalence of 52.9% using the PrioCHECK^®^ ELISA. Furthermore, a study in Spain by Castillo-Cuenca et al. [30] revealed a seroprevalence of 25.9% in fattening pigs and sows. The utilization of the same ELISA kit in these findings contributes to the consistency and reliability of the reported seroprevalence rates across diverse geographical locations. Recent reports from neighboring countries of northeastern India, namely China and Myanmar, which share international boundaries with India, reveal seroprevalences of *T*. *gondii* in pigs as 20.85% [31] and 18.4% [32], respectively. In India, *T*. *gondii* is circulating among the human population, including blood donors and pregnant women, with multiple reports available in the public domain [5, 33, 34]. Additionally, three reports document the seroprevalence of *T*. *gondii* in veterinarians, slaughterhouse workers, pet keepers, and farmers in Northern India [35], Central India [36], and Northeastern India [37]. This emphasizes the significance of *T*. *gondii* as a zoonotic pathogen and highlights the occupational risks faced by animal rearers and handlers.

Similarly, reports of *Trichinella* spp. in the livestock and wildlife populations in India, including Northeastern India, are limited [17, 38, 39]. In Central India, Kumar et al. [17] documented a larval prevalence of 0.69% for *Trichinella* spp. in the diaphragms of slaughtered pigs in an abattoir. In Assam (Northeastern India), Konwar et al [38] reported a seroprevalence of 2.87% for *Trichinella* spp. Acheenta et al. [40] found anti-*Trichinella* antibodies in porcine sera from Assam, Meghalaya, Arunachal Pradesh, Mizoram, and Tripura at rates 0.27%, 0.41%, 0%, 0%, and 0%, respectively. Consistent with these earlier reports from India, the present study also revealed a seroprevalence of 0.75% for *Trichinella* spp. in pigs from Northeastern India. Specifically in Manipur and Arunachal Pradesh, 2.5% and 1.25% of the samples, respectively, tested positive for *Trichinella* antibodies. No seropositive samples were identified in Meghalaya, Mizoram, and Nagaland. Multiple human case reports of trichinellosis causing pyomyositis and osteomyelitis have been documented across India [41, 42]. In Uttarakhand, a northern state of India, multiple outbreaks of trichinellosis occurred, with 70 suspected cases and 11 mortalities reported following the consumption of undercooked or raw wild boar meat [43]. Nehra et al. [39] also described the molecular detection of *T*. *spiralis* in a deceased leopard in Uttarakhand. Therefore, although a rare zoonosis, trichinellosis poses a threat to the Indian population, especially among those who regularly consume pork and bush meat.

In this study, *T*. *gondii* seropositivity in the tested pigs was strongly correlated with certain risk factors. Significantly higher seropositivity was observed in pigs sampled from three states, namely Nagaland (OR 16.23, 95% CI 7.66–36.65), Mizoram (OR 5.17, 95% CI 2.63–10.50), and Meghalaya (OR 2.68, 95% CI 1.36–5.40). Similar regional variations were noted in a prior study in Spain, possibly influenced by factors such as climate and cultural practices [44]. Age emerged as a significant factor in seropositivity, with pigs aged between 13–24 months (OR 2.24, 95% CI 1.23–4.12) and those above 24 months (OR 13.07, 95% CI 5.27–37.69) showing a higher likelihood of seropositivity. Similar age-related trends have been observed in earlier studies, indicating prolonged exposure to oocysts and carcasses of other animals and rodents in older pigs [45–48]. Farms maintaining local or non-descript pig breeds (OR 1.90, 95% CI 1.15–3.17) exhibited higher seropositivity compared to farms maintaining crossbred and exotic pigs. This difference might be attributed to better hygienic management practices and disinfection on farms with improved breeds. Additionally, farms operating for breeding purposes (OR 5.60, 95% CI 3.01–11.04) were significantly associated with high seropositivity, aligning with similar observations in earlier studies where breeding farms and farms not following an all-in-all-out production system displayed higher *T*. *gondii* seroprevalence [44, 49]. Although not statistically significant, higher seropositivity was noticed among sows compared to boars, a common scenario in breeding farms. This could be attributed to increased age-related contact and the lifelong persistence of antibodies [44, 47]. Farms with a history of abortion and stillbirth (OR 1.90, 95% CI 1.12–3.25) exhibited a statistically significant correlation with *T*. *gondii* seropositivity, likely due to the persistence of *T*. *gondii* oocysts in the farm premises. In the final multivariate regression model, *T*. *gondii* seroprevalence was strongly associated with age and exposure to cats. The presence of cats in farm premises has consistently been identified as a significant risk factor in numerous previous studies [44, 50, 51]. Given that cats are the definitive hosts for *T*. *gondii* and can shed over 20 million oocysts during a three-week infection cycle, they substantially contribute to the environmental contamination of pig farms [48].

The present study has a few limitations. Notably, genetic characterization or diversity analysis of *T*. *gondii* was not conducted, as the haplotypes circulating in the Indian livestock population remains unknown. Additionally, the limited number of serum samples possessing *Trichinella* spp. antibodies (n = 3) hindered the ability to conduct risk factor analysis using univariate and multivariate logistic regression. Addressing these limitations would require a larger sample size, and these considerations should guide future studies.

## Conclusions

This study offers detailed insight into the seroepidemiological status of *T*. *gondii* and *Trichinella* spp. in pigs in Northeastern India. While toxoplasmosis can be considered highly prevalent, *Trichinella* spp. infection was found to be of low prevalence (below 1%). Significant risk factors associated with the seroprevalence of *T*. *gondii* included age above 24 months, exposure to cats, and farms operating for breeding purposes. The high prevalence of toxoplasmosis in pigs may play a crucial role in disease transmission to humans, particularly among farmers, abattoir workers, and pork consumers through direct and indirect contact with infected pigs and meat. Advocating for public education and awareness campaigns regarding the risks of toxoplasmosis and preventive measures is crucial for preventing and controlling the spread of the disease. Generating baseline data on the seroprevalence of neglected zoonotic diseases can be helpful to livestock, human, and environmental health authorities. In the One Health era, knowledge sharing between sectors becomes paramount to curbing the burden of emerging infectious diseases through informed planning and evidence-based programmes. The study’s coverage of the pig population in five Northeastern Indian states, sharing porous international boundaries with China, Myanmar, Bhutan, and Bangladesh, provides findings that may be valuable to their authorities, given the porous nature of the borders and the common clandestine movement of pigs and pig products.

## Supporting information

S1 Data (XLSX)

S2 Data (XLSX)
